# Supplementary material for: A miR-327–FGF10–FGFR2-mediated autocrine signaling mechanism controls white fat browning
Source: Nat Commun. 2017 Dec 12;8:2079. doi: 10.1038/s41467-017-02158-z (PMC5727036; doi:10.1038/s41467-017-02158-z)
Supplement: Supplementary file 1 — Supplementary Information [file 41467_2017_2158_MOESM1_ESM.pdf]

Supplementary information

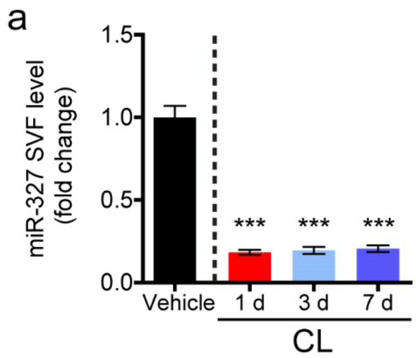

Supplementary Figure 1 | CL-316243 stimulation downregulates miR-327 in WAT-SVF

(**a**) qPCR analysis of miR-327 in visWAT-SVFs of 1-, 3- and 7-day CL-316243- treated C57BL/6 mice compared to vehicle treated controls. Sno-202 served as internal control (n = 5 samples per group).

a

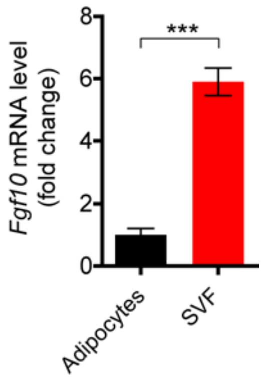

Supplementary Figure 2 | *Fgf10* mRNA is predominantly expressed by non-adipocytes in WAT. (**a**) qPCR analysis of WAT-SVFs compared to the WAT-adipocyte fractions isolated from C57BL/6 mice. *Actb* served as an internal control (n = 5 samples per group). n.s., not significant. *P<0.05, **P<0.01, and ***P<0.001 by Student's *t*-test. Data presented as mean ± s.e.m.

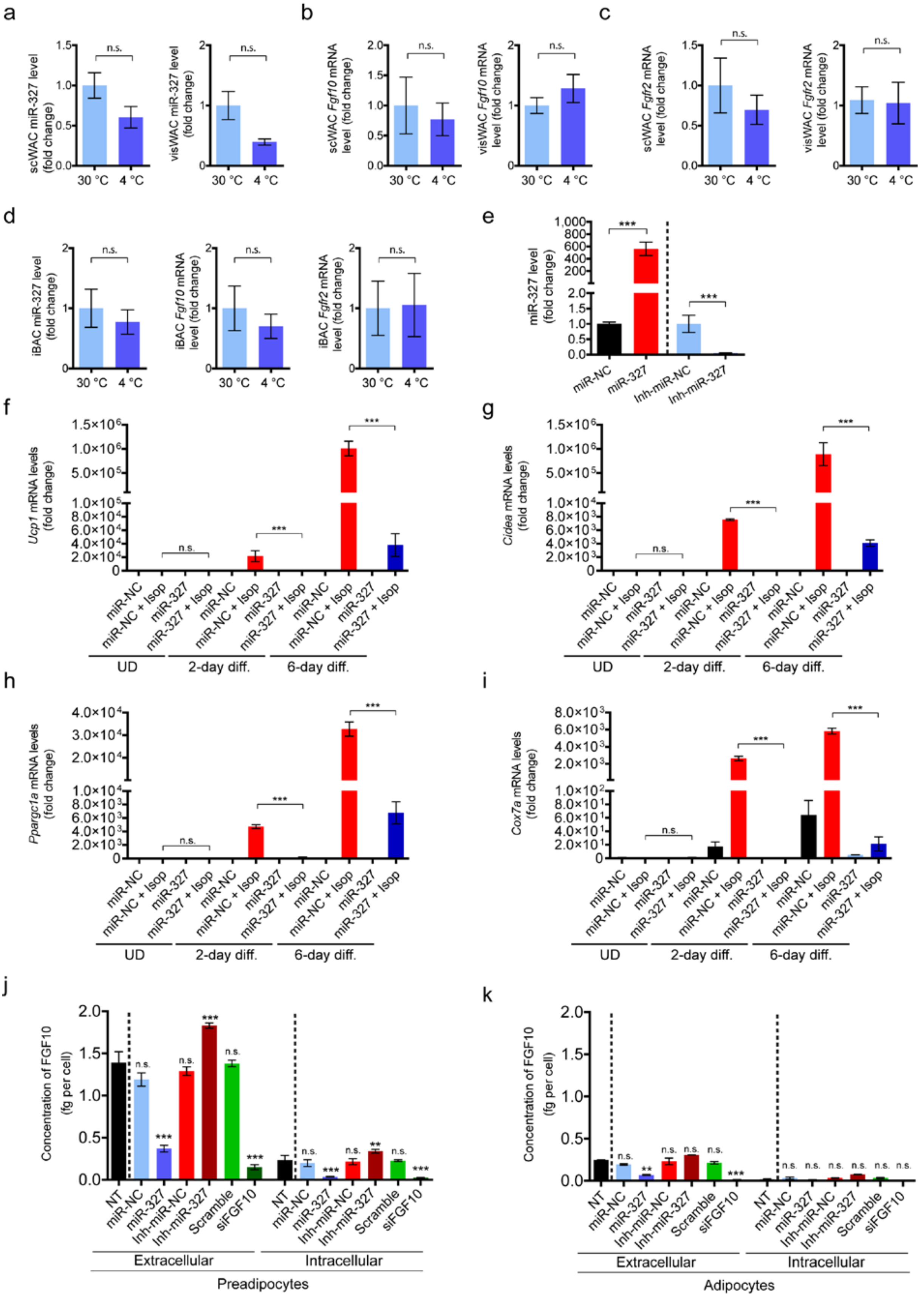
a
scWAC miR-327 level (fold change)
visWAC miR-327 level (fold change)
b
scWAC Fgf10 mRNA level (fold change)
visWAC Fgf10 mRNA level (fold change)
c
scWAC Fgfr2 mRNA level (fold change)
visWAC Fgfr2 mRNA level (fold change)
n.s.
30 °C
4 °C
d
iBAC miR-327 level (fold change)
iBAC Fgf10 mRNA level (fold change)
iBAC Fgfr2 mRNA level (fold change)
e
miR-327 level (fold change)
miR-NC
miR-327
Inh-miR-NC
Inh-miR-327
f
Ucp1 mRNA levels (fold change)
g
Cidea mRNA levels (fold change)
h
Ppargc1a mRNA levels (fold change)
i
Cox7a mRNA levels (fold change)
miR-NC
miR-NC + Isop
miR-327
miR-327 + Isop
UD
2-day diff.
6-day diff.
j
Concentration of FGF10 (fg per cell)
NT
miR-NC
miR-327
Inh-miR-NC
Inh-miR-327
Scramble
siFGF10
Extracellular
Intracellular
Preadipocytes
k
Concentration of FGF10 (fg per cell)
Extracellular
Intracellular
Adipocytes

Supplementary Figure 3 | Effects of browning and miR-327 alterations on miR-327, *Fgf10* and *Fgfr2* RNA levels, browning factors and FGF10 protein levels. (**a-d**) qPCR analysis of miR-327, *Fgf10* and *Fgfr2* in primary white adipocytes (WAC) and brown adipocytes (BAC) derived from scWAT, visWAT and iBAT from 1-week-4 °C-exposed C57BL/6 mice relative to the 30 °C control group. Sno-202 or *Actb* served as internal controls (n = 5 samples per group). (**e**) qPCR analysis of miR-327 levels in 3T3-L1 preadipocytes treated with miR-327 mimics or inhibitors compared to respective controls. Sno-202 served as internal control (n = 5 samples per group). (**f-i**) qPCR analysis of *Ucp1, Cidea, Ppargc1a,* and *Cox7a* in undifferentiated (UD), 2-day differentiated (2-day diff.) and 6-day differentiated (6-day diff.) 3T3-L1 cells treated with miR-NC, miR-NC plus isoproterenol (miR-NC + Isop) for 4h, miR-327, and miR-327 plus isoproterenol (miR-NC + Isop.). mRNA levels were normalized to the miR-NC group and *Actb* served as an internal control (n = 5 samples per group). (**j, k**) ELISA analysis of extracellular and intracellular FGF10 protein levels in 3T3-L1 preadipocytes and differentiated adipocytes receiving non-treatment (NT) or treatment prior to differentiation with miR-NC, miR-327, Inh-miR-327, Scramble, or siFGF10. Extracellular FGF10 concentrations were determined using 72 h-conditioned medium (n = 5 samples per group). n.s., not significant. *P<0.05, **P<0.01, and ***P<0.001 by Student's *t*-test. Data presented as mean ± s.e.m.

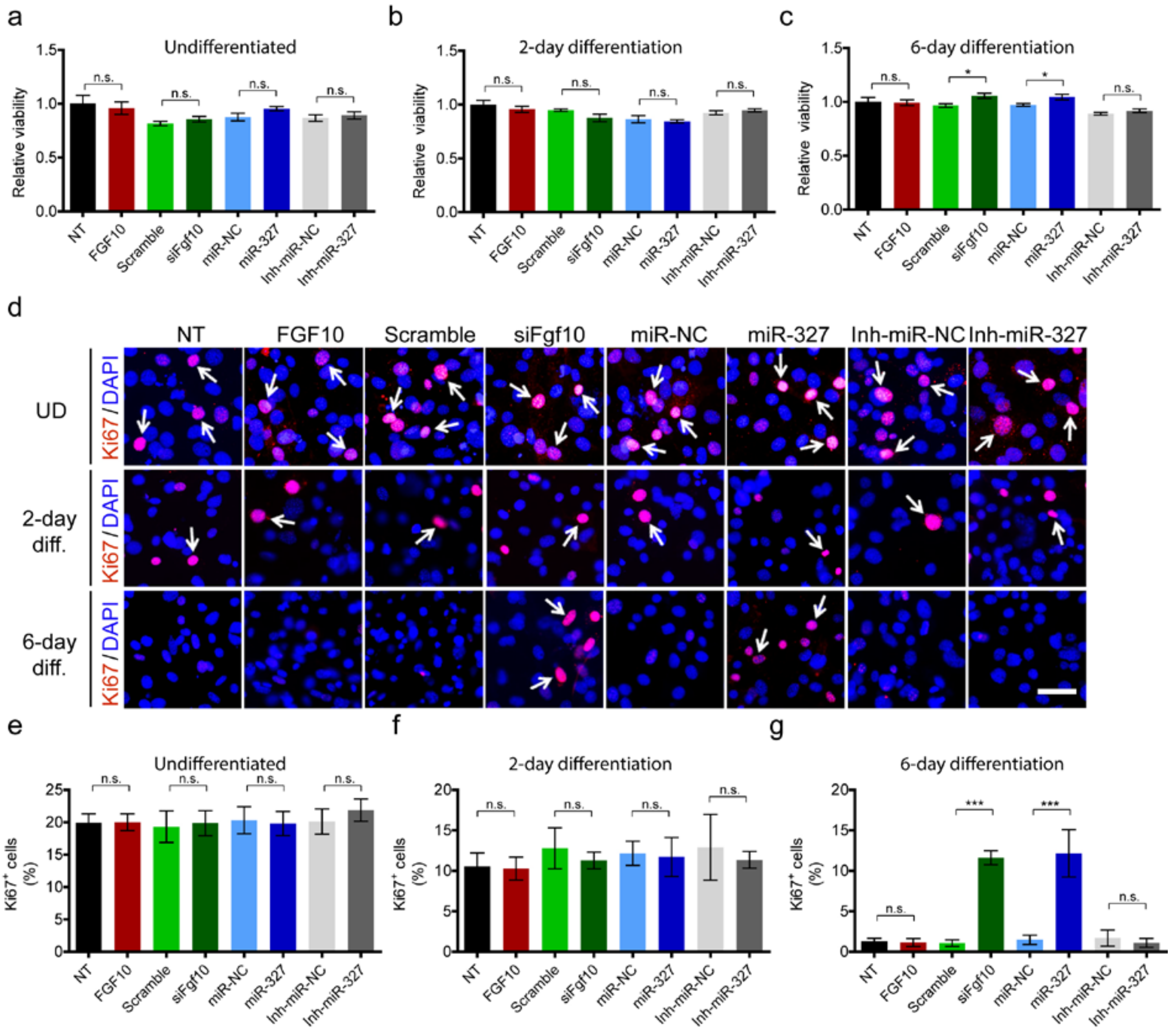

Supplementary Figure 4 | Proliferation of preadipocytes under various treatment conditions. (**a-c**) Proliferation of undifferentiated (UD), 2-day differentiated (2-day diff.) and 6-day differentiated (6-day diff.) 3T3-L1 cells receiving recombinant FGF10, siFgf10, miR-327 mimic or miR327 inhibitor treatment. Proliferating cells were normalized to the non-treated (NT) controls (n = 8 samples per group). (**d-g**) Immunohistochemical analysis and quantification of Ki67+ proliferating cells. DAPI was used to stain cell nuclei. Arrows point to proliferating cells, Scale bar, 100 μm, >30 adipocytes per field; n = 10 random fields. n.s., not significant. *P<0.05, **P<0.01, and ***P<0.001 by Student's *t*-test. Data presented as mean ± s.e.m.

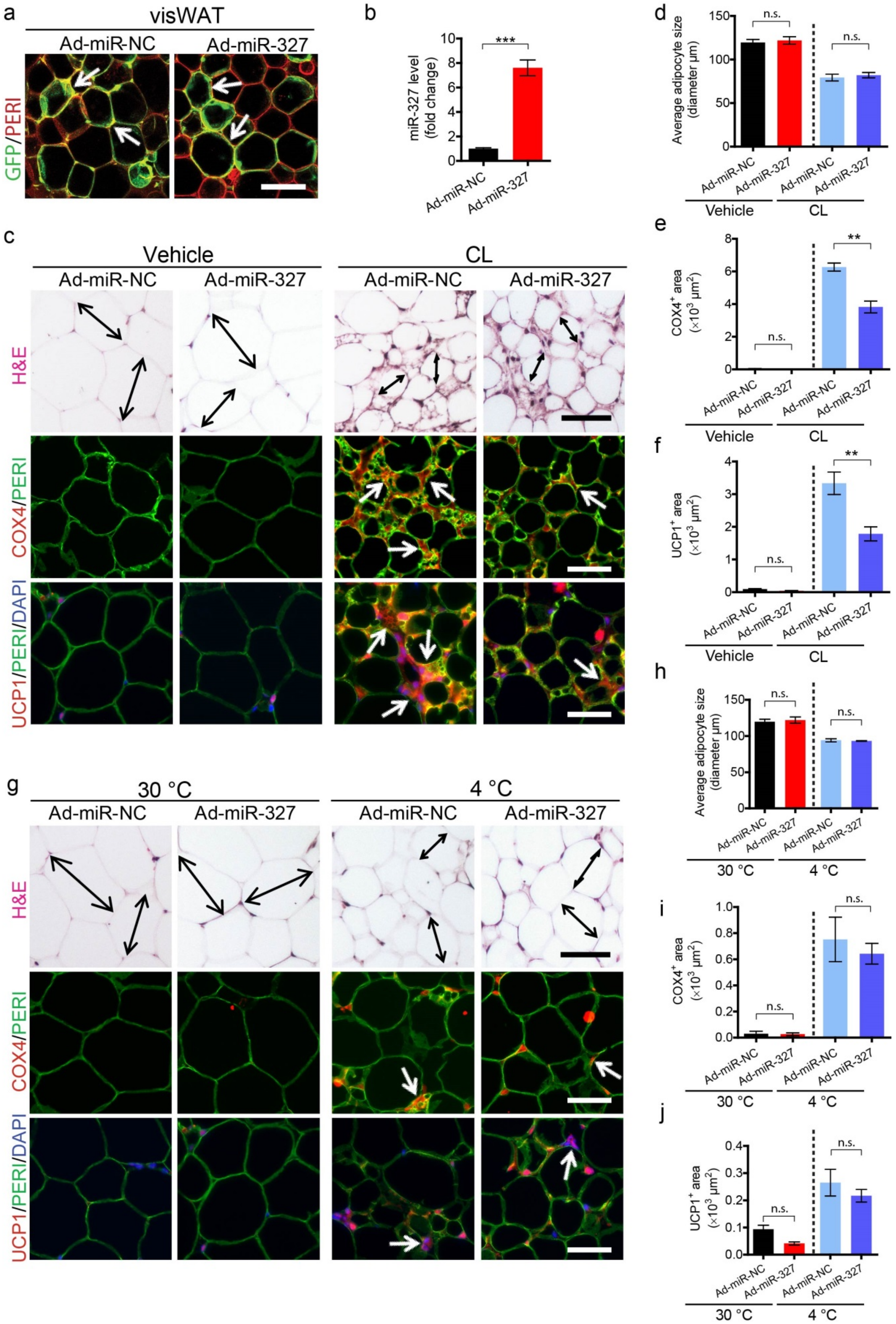

a
visWAT
Ad-miR-NC
Ad-miR-327
GFP/PERI
b
miR-327 level (fold change)
***
Ad-miR-NC
Ad-miR-327
c
Vehicle
CL
Ad-miR-NC
Ad-miR-327
H&E
COX4/PERI
UCP1/PERI/DAPI
d
Average adipocyte size (diameter μm)
n.s.
e
COX4+ area (×10³ μm²)
**
f
UCP1+ area (×10³ μm²)
g
30 °C
4 °C
h
i
j

1

Supplementary Figure 5 | Ad-miR-327 inhibits WAT browning. (**a**) Histological analysis of GFP$^+$ cells in visWAT transfected with a control adenovirus (Ad-miR-NC) or an adenovirus expressing miR-327 (Ad-miR-327). Perilipin (PERI) was used to identify adipose tissues. Arrows point to GFP$^+$ cells. (**b**) qPCR analysis of miR-327 expression in Ad-miR-327 compared to Ad-miR-NC. Sno-202 served as internal control (n = 6 samples per group). (**c, g**) Histological analysis of adipocyte morphology (H&E), adipocytes (PERI), mitochondria (COX4) and uncoupling protein (UCP1) in (**c**) 5-day CL-316243 treated visWAT compared to vehicle treated control. (**g**) 2-week 4 °C treated visWAT compared to 30 °C control. Double-headed arrows mark adipocyte diameter. Arrows point to respective positive signals. (**d-f** and **h-j**) Quantifications of adipocyte size and positive signals of COX4 and UCP1 in (**d-f**) CL-316243- and vehicle-, and (**h-j**) 30 °C- and 4 °C- treated visWATs (>30 adipocytes per field; n = 10 random fields; n =6 mice per group). Immunodeficient NSG mice were used for all experiments in this figure. Scale bars, 100 µm. n.s., not significant. **P<0.01, and ***P<0.001 by Student's t-test. Data presented as mean ± s.e.m.

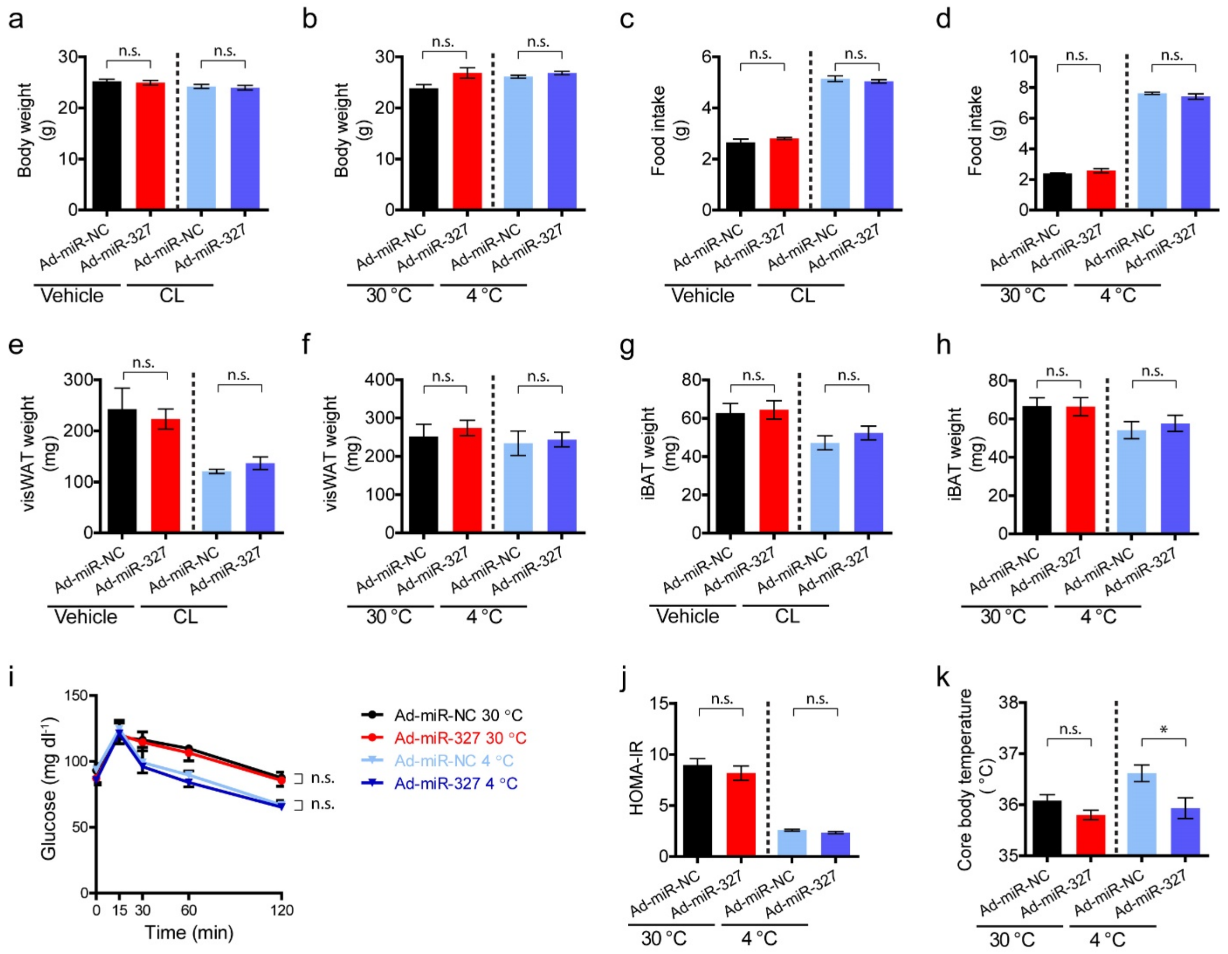

Supplementary Figure 6 | Global metabolic changes of miR-327-treated mice (**a-h**) Body weight, food intake, visWAT weight, and iBAT weight of NSG mice treated with Ad-miR-NC or Ad-miR-327, followed by 5-day CL-316243 treatment or 2-week 4 °C exposure (n = 12 mice per group). (**i**) Glucose tolerance test (GTT) of Ad-miR-NC- or Ad-miR-327-treated NSG mice under 2-week 30 °C or 4 °C exposure (n = 6-8 mice per group). (**j**) Homeostatic model assessment of insulin resistance (HOMA-IR) of Ad-miR-NC- or Ad-miR-327-treated NSG mice exposed for under 2-week 30 °C or 4 °C exposure (n = 6 mice per group). (**k**) Core body temperature of Ad-miR-NC- or Ad-miR-327-treated NSG mice under 2-week 30 °C or 4 °C exposure (n = 12 mice per group). n.s., not significant. *P<0.05, **P<0.01, and ***P<0.001 by Student's *t*-test. Data presented as mean ± s.e.m.

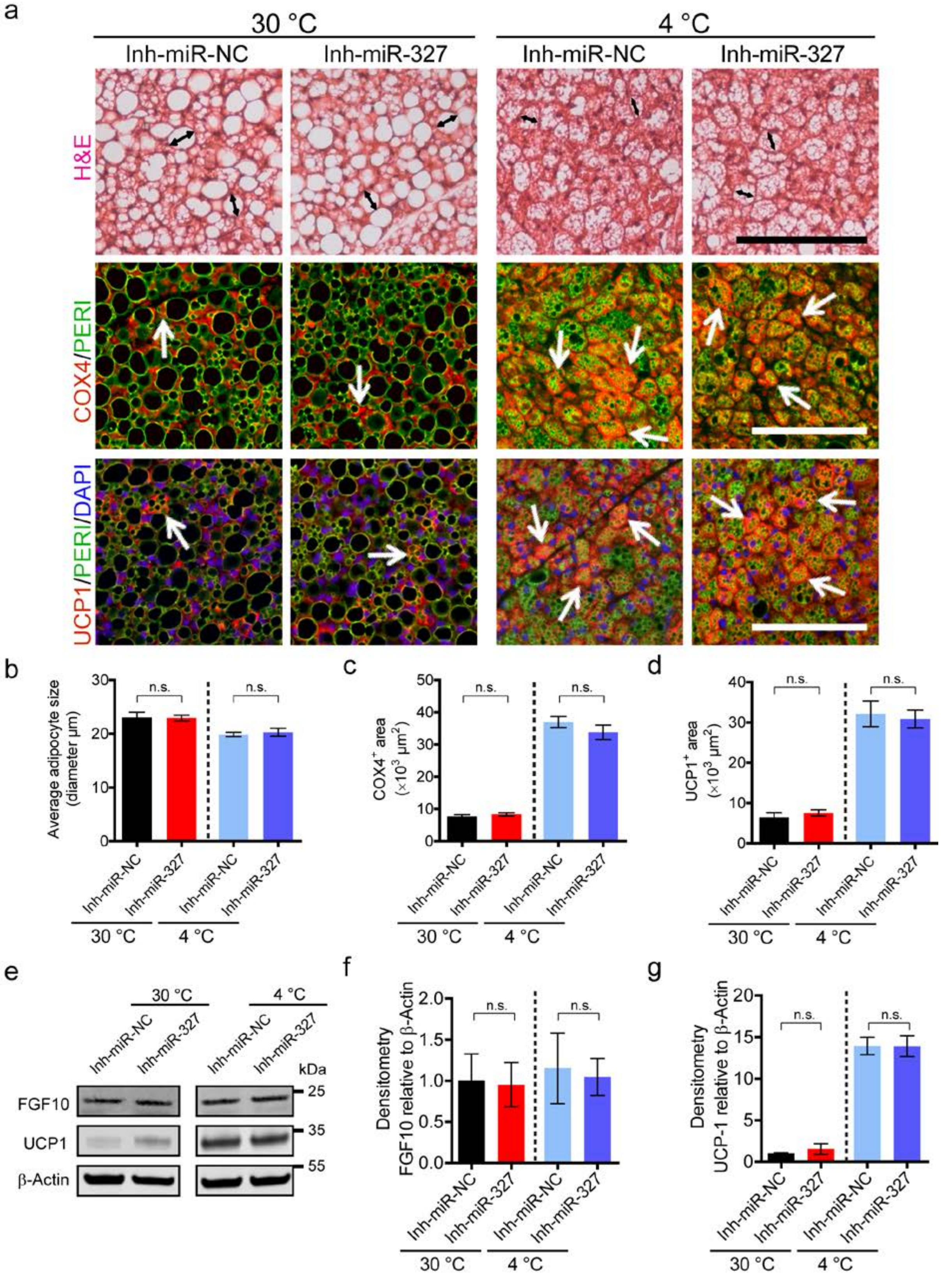
a
30 °C
4 °C
Inh-miR-NC
Inh-miR-327
Inh-miR-NC
Inh-miR-327
H&E
COX4/PERI
UCP1/PERI/DAPI
b
Average adipocyte size (diameter µm)
n.s.
n.s.
c
COX4+ area (×10^3 µm^2)
d
UCP1+ area (×10^3 µm^2)
e
FGF10
UCP1
β-Actin
kDa
25
35
55
f
Densitometry FGF10 relative to β-Actin
g
Densitometry UCP-1 relative to β-Actin

Supplementary Figure 7 | Inhibition of miR-327 does not affect BAT. (**a**) Histological analysis of adipocyte morphology (H&E), adipocytes (PERI), mitochondria (COX4) and uncoupling protein 1 (UCP1) in Inh-miR-NC- and Inh-miR-327-treated iBAT under 2-week 30 °C or 4 °C exposure. Double-headed arrows mark adipocyte diameters. Arrows point to respective positive signals. (**b-d)** Quantifications of adipocyte size and positive signals of COX4 and UCP1 in Inh-miR-NC- and Inh-miR-327-treated iBAT under 2-week 30 °C or 4 °C exposure (>30 adipocytes per field; n = 10 random fields; n = 4 mice per group). (**e-g**) Western immunoblot analysis and quantification of FGF10 and UCP1 in Inh-miR-NC- and Inh-miR-327-treated iBAT under 2-week 30 °C or 4 °C exposure. FGF10 and UCP1 protein levels were quantified as densitometric signals and normalized to β-Actin (n = 4 samples per group). Scale bars, 100 μm. kDa, kilodalton. n.s., not significant. *P<0.05, **P<0.01, and ***P<0.001 by Student's *t*-test. Data presented as mean ± s.e.m.

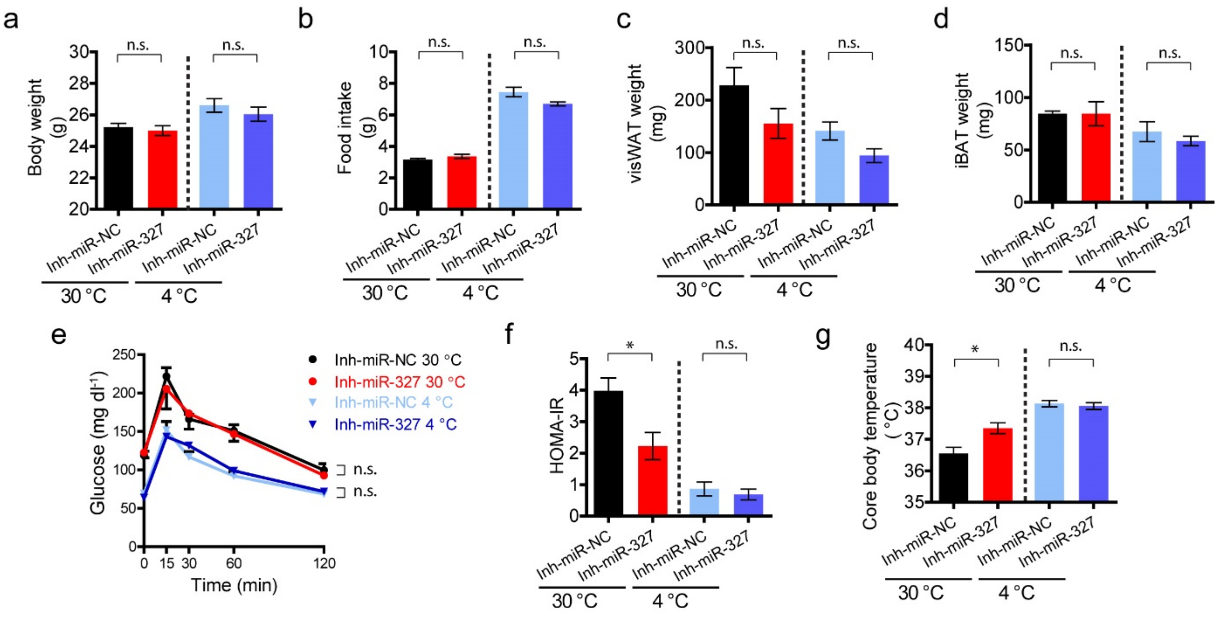

Supplementary Figure 8 | Global metabolic changes of miR-327 inhibitor-treated mice. (**a-d**) Body weight, food intake, visWAT weight, and iBAT weight of C57BL/6 mice treated with Inh-miR-NC or Inh-miR-327, followed by 2-week 4 °C or 30 °C exposure (n = 12 mice per group). (**e**) Glucose tolerance test (GTT) of Inh-miR-NC- or Inh-miR-327-treated C57BL/6 mice under 2-week 30 °C or 4 °C exposure (n = 6-8 mice per group). (**f**) Homeostatic model assessment of insulin resistance (HOMA-IR) of Inh-miR-NC- or Inh-miR-327-treated mice under 2-week 30 °C or 4 °C exposure (n = 6 mice per group). (**g**) Core body temperature of Inh-miR-NC- or Inh-miR-327-treated mice under 2-week 30 °C or 4 °C exposure (n = 12 mice per group). n.s., not significant. *P<0.05, **P<0.01, and ***P<0.001 by Student's *t*-test. Data presented as mean ± s.e.m.

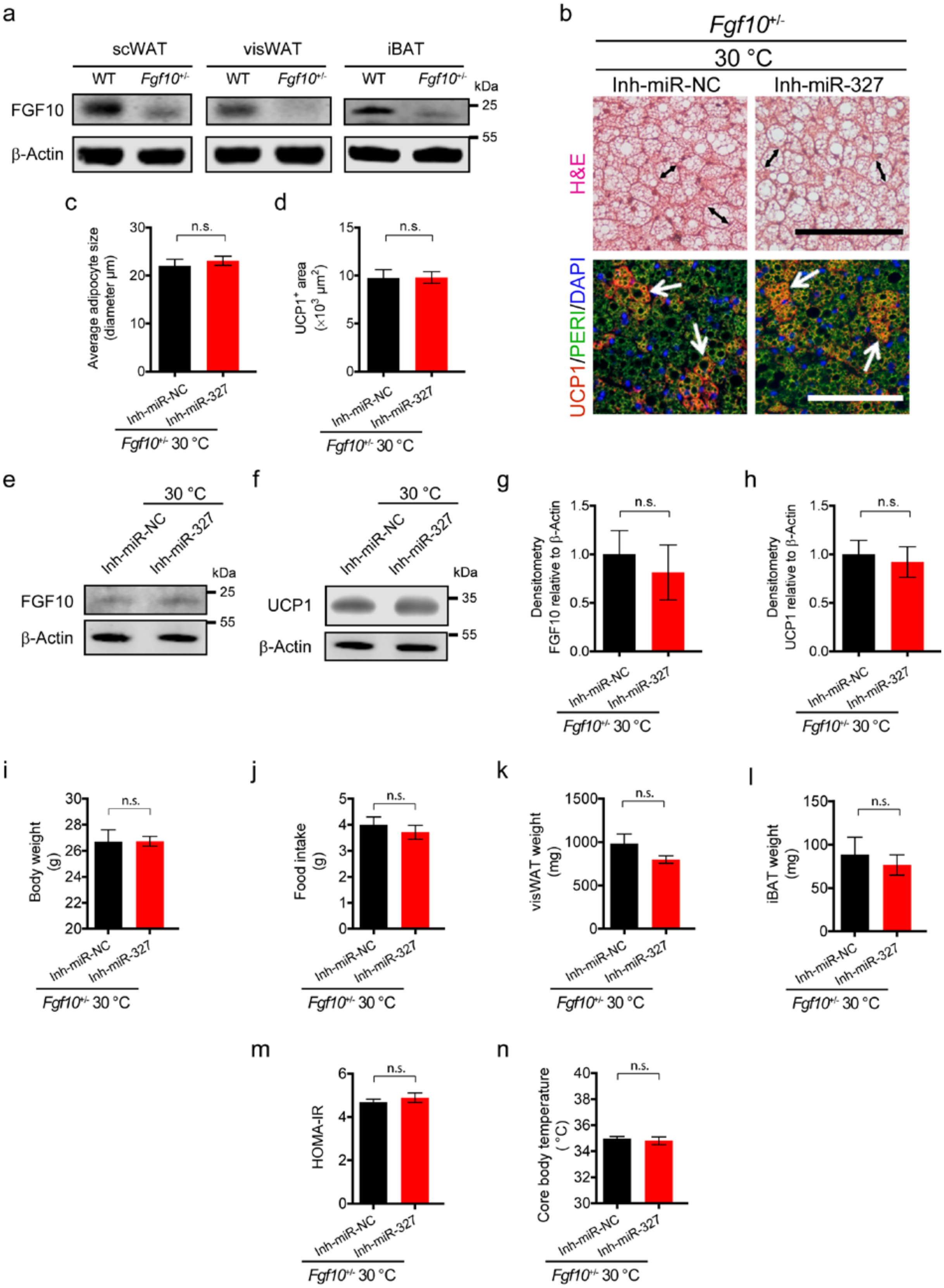
a
scWAT
visWAT
iBAT
WT
Fgf10+/-
kDa
FGF10
β-Actin
25
55
b
Fgf10+/-
30 °C
Inh-miR-NC
Inh-miR-327
H&E
UCP1/PERI/DAPI
c
Average adipocyte size (diameter μm)
n.s.
Fgf10+/- 30 °C
d
UCP1+ area (×10³ μm²)
e
f
UCP1
35
g
Densitometry FGF10 relative to β-Actin
h
Densitometry UCP1 relative to β-Actin
i
Body weight (g)
j
Food intake (g)
k
visWAT weight (mg)
l
iBAT weight (mg)
m
HOMA-IR
n
Core body temperature (°C)

Supplementary Figure 9 | Attenuation of miR-327 inhibition-triggered global metabolic phenotype in *Fgf10*$^{+/-}$ mice. (**a**) Western immunoblot analysis of FGF10 in scWAT, visWAT and iBAT of *Fgf10*$^{+/-}$ mice compared to those of WT mice. (**b**) Histological analysis of adipocyte morphology (H&E), adipocytes (PERI) and uncoupling protein 1 (UCP1) in iBAT isolated from Inh-miR-NC- and Inh-miR-327-treated 2-week-30 °C- exposed *Fgf10*$^{+/-}$ mice. Double-headed arrows mark adipocyte diameters. Arrows point to respective positive signals. (**c-d)** Quantifications of adipocyte size and positive signals of UCP1 in Inh-miR-NC- and Inh-miR-327-treated 2-week 30 °C-exposed iBAT derived from *Fgf10*$^{+/-}$ mice (>30 adipocytes per field; n = 10 random fields). (**e-h**) Western immunoblot analysis and quantification of FGF10 and UCP1 proteins in Inh-miR-NC- and Inh-miR-327-treated *Fgf10*$^{+/-}$ iBAT under 2-week 30 °C exposure. FGF10 and UCP1 protein levels were quantified as densitometric signals and normalized to β-Actin (n = 5 samples per group). (**i-n**) Body weight, food intake, visWAT weight, iBAT weight, HOMA-IR, and core body temperature of *Fgf10*$^{+/-}$ mice treated with Inh-miR-NC or Inh-miR-327 under 2-week 30 °C or 4 °C exposure (n = 5-8 samples per group). Scale bars, 100 μm. kDa, kilodalton. n.s., not significant. *P<0.05, **P<0.01, and ***P<0.001 by Student's *t*-test. Data presented as mean ± s.e.m.

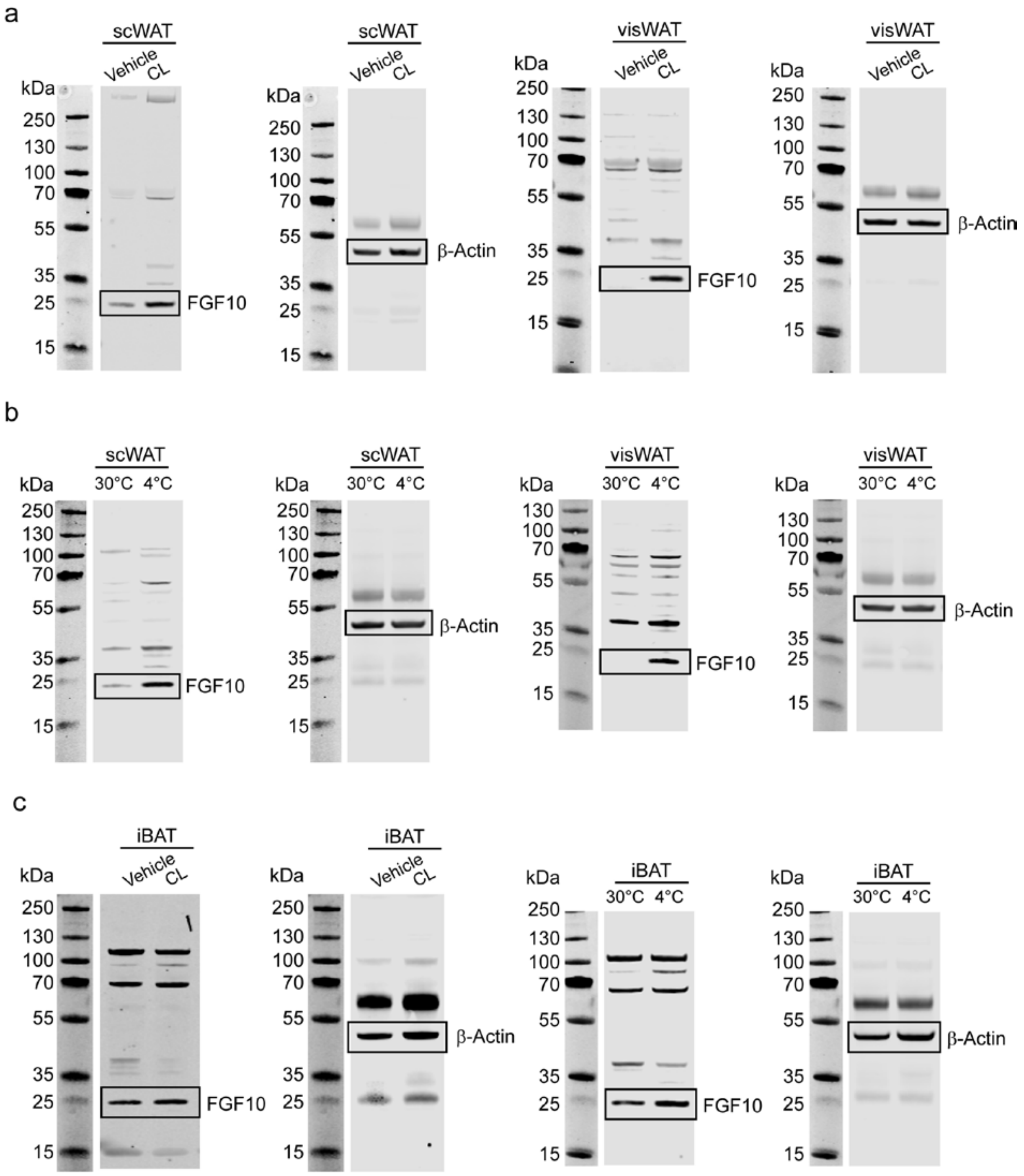

Supplementary Figure 10 | Full gel scans for Fig 3a, 3b and 3c. (**a**) Gel scan for Fig. 3a. (**b**) Gel scan for Fig. 3b. (**c**) Gel scan for Fig. 3c.

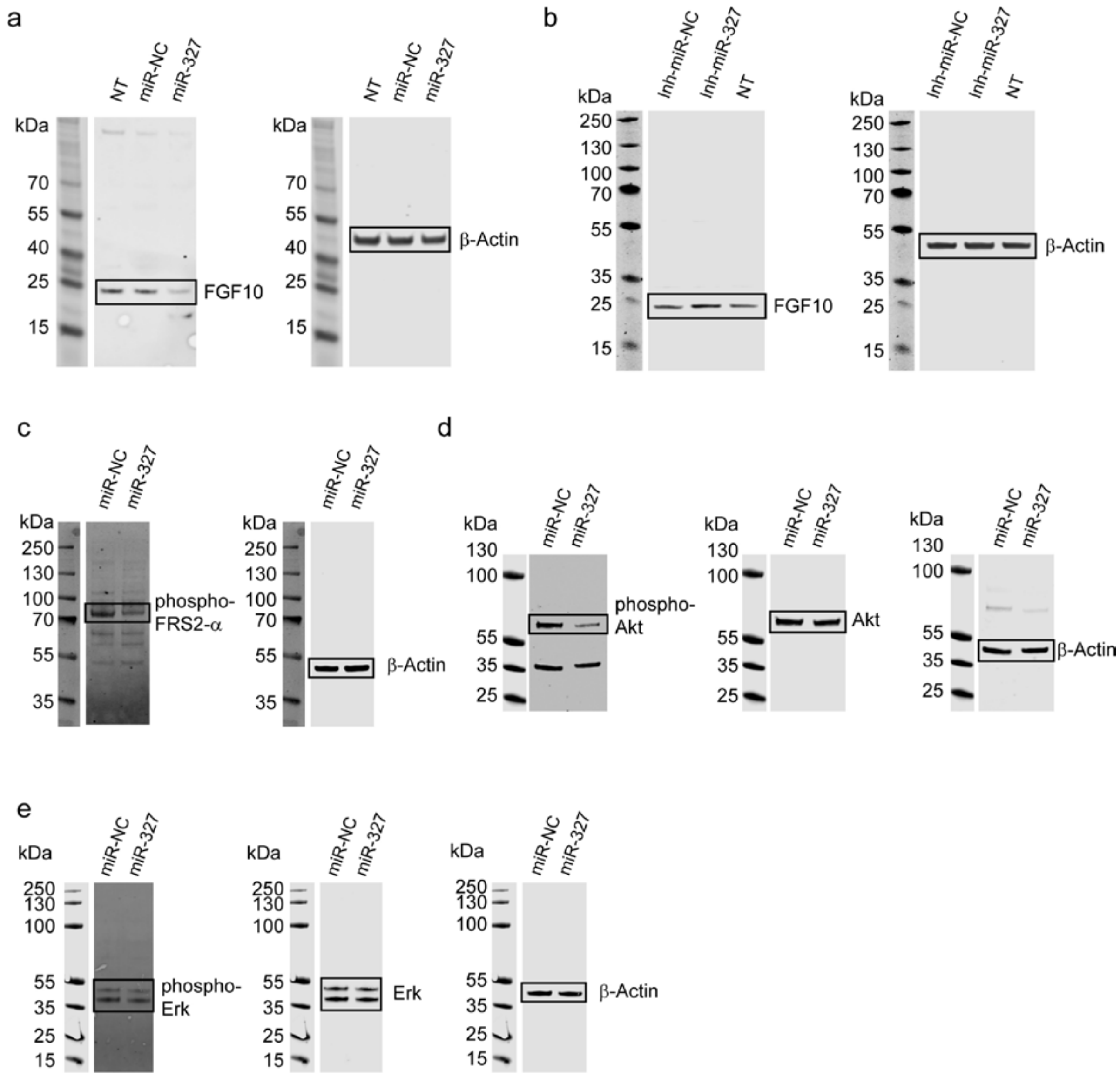

Supplementary Figure 11 | Full gel scans for Fig 4b, 4e, 4g, 4i and 4k (**a**) Gel scan for Fig. 4b. (**b**) Gel scan for Fig. 4e. (**c**) Gel scan for Fig. 4g. (**d**) Gel scan for Fig. 4i. (**e**) Gel scan for Fig. 4k.

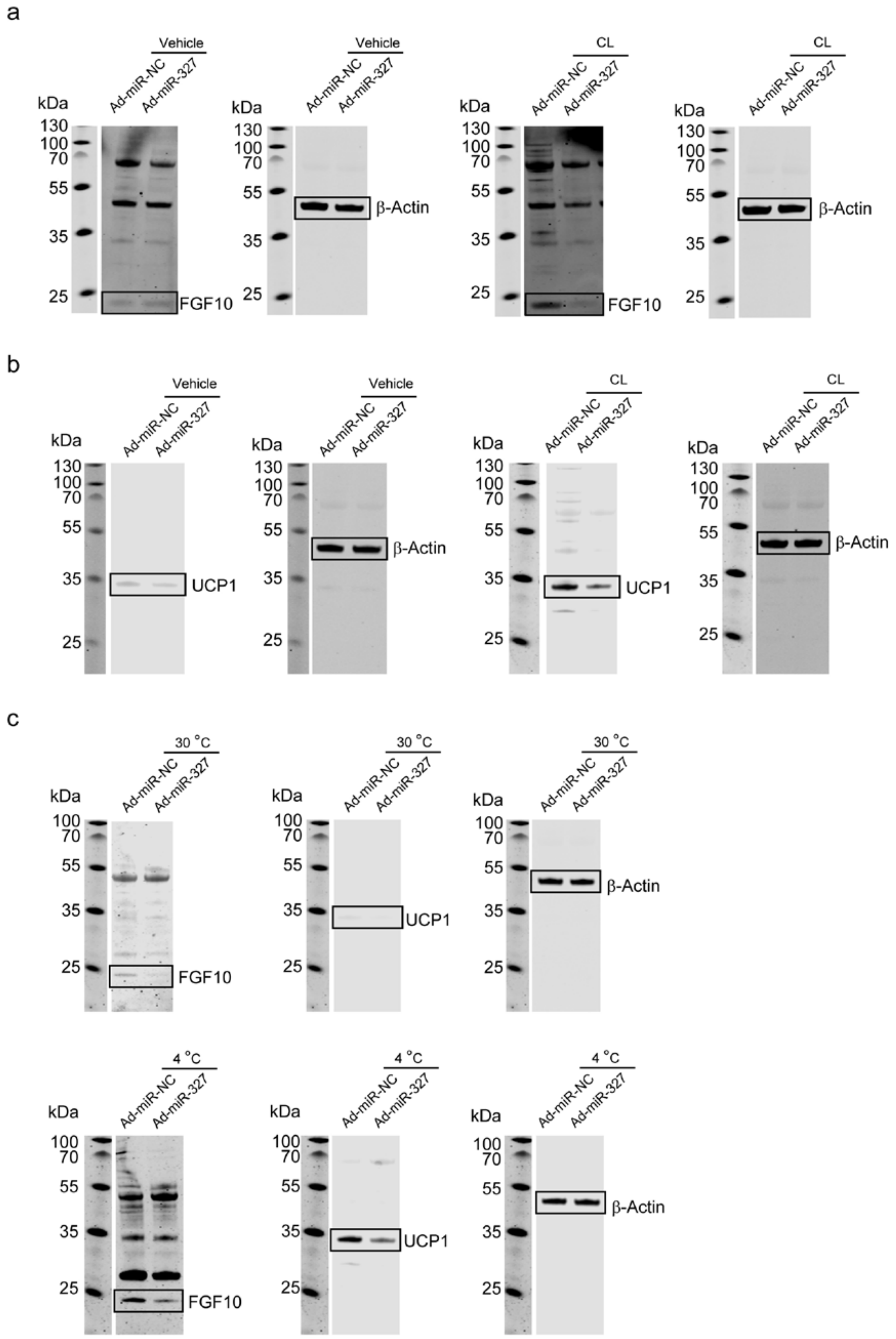

Supplementary Figure 12 | Full gel scans for Fig 8a, 8b and 8c (**a**) Gel scan for Fig. 8a. (**b**) Gel scan for Fig. 8b. (**c**) Gel scan for Fig. 8c.

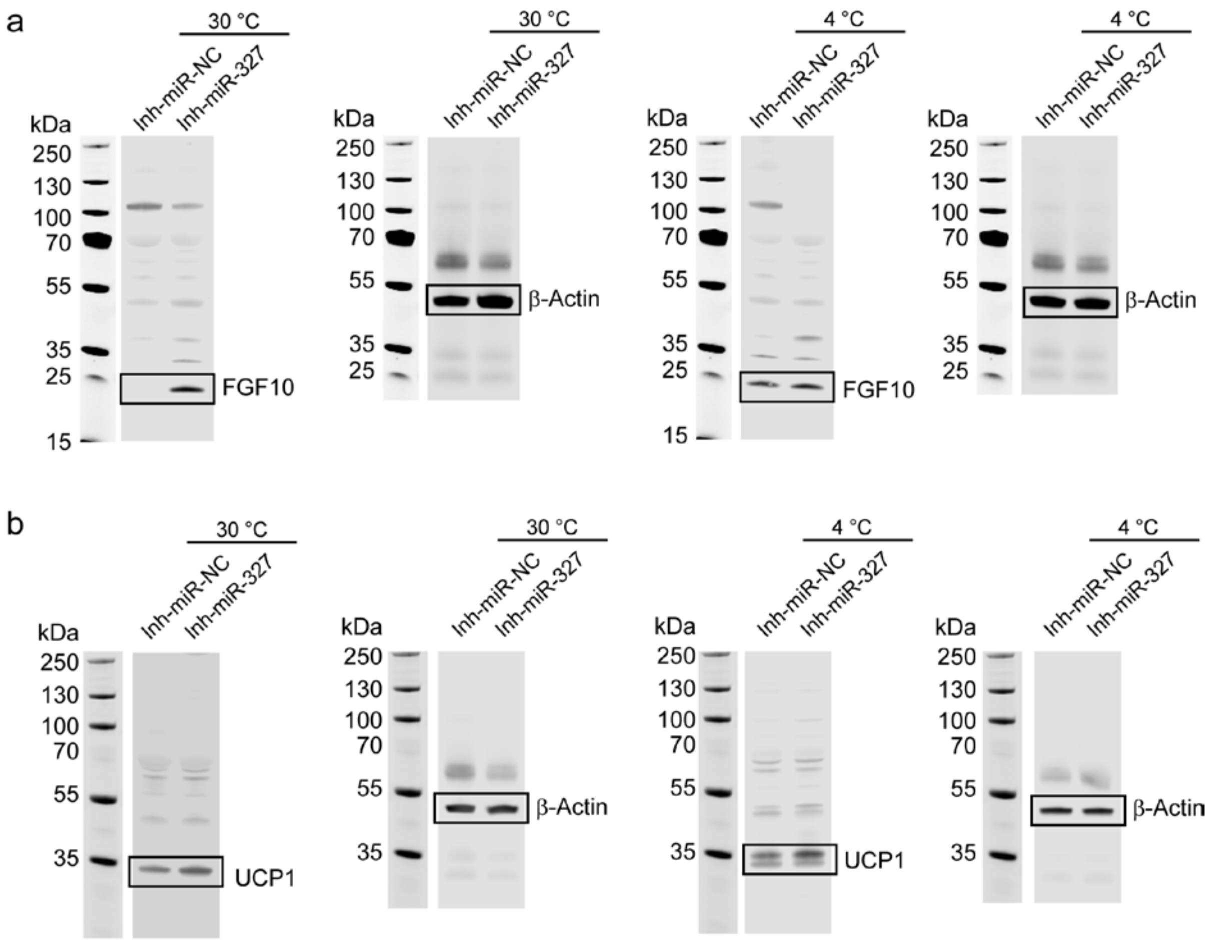

Supplementary Figure 13 | Full gel scans for Fig 9e and 9f (**a**) Gel scan for Fig. 9e. (**b**) Gel scan for Fig. 9f.

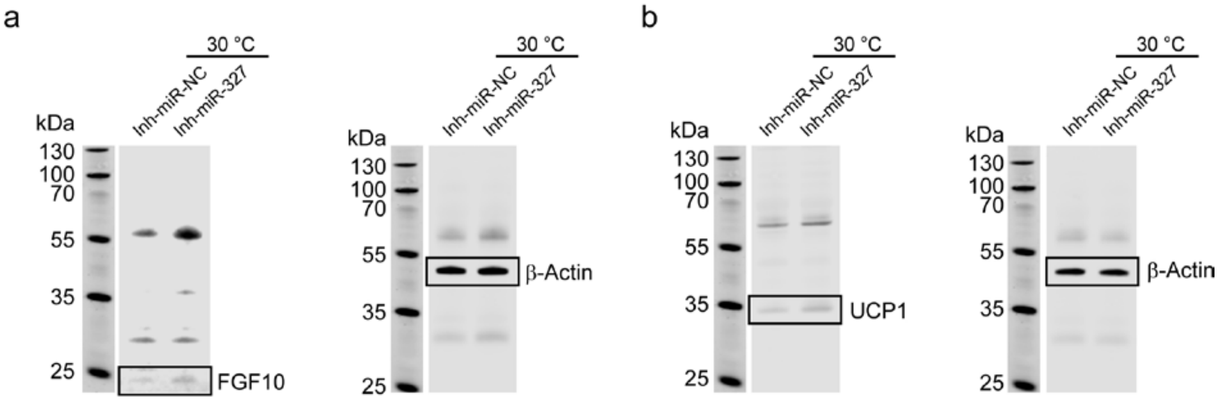

Supplementary Figure 14 | Full gel scans for Fig 10e and 10f (**a**) Gel scan for Fig. 10e. (**b**) Gel scan for Fig. 10f.

Supplementary Table 1 | Primer sequences

Gene	Forward primer sequence	Reverse primer sequence
**qPCR primers**		
Fgf10	5’-CCGACACCACCAGTTCCTAC-3’	5’-CTTTGACGGCAACAACTCCG-3’
Actin	5’-AGGCCCAGAGCAAGAGAGG-3’	5’-TACATGGCTGGGGTGTTGAA-3’
Pparg	5’-GTGCCAGTTTCGATCCGTAGA-3’	5’-GGCCAGCATCGTGTAGATGA-3’
Prdm16	5’-CAGCACGGTGAAGCCATTC-3’	5’-GCGTGCATCCGCTTGTG-3’
Ppargc1a	5’-AGCCGTGACCACTGACAACGAG-3’	5’-GCTGCATGGTTCTGAGTGCTAAG-3’
Adipoq	5’-CTTTCATGTACACCGTGATGTG-3’	5’-ACCTCTCCTGTTCCTCTTAATCC-3’
Cebpa	5’-TGGAGACGCAACAGAAGGTG-3’	5’-CAGCCTAGAGATCCAGCGAC-3’
Cebpb	5’-GGGGTTGTTGATGTTTTTGGT-3’	5’-TCGAAACGGAAAAGGTTCTCA-3’
Cebpg	5’-AATTGGCCCCAAAGAGCCTG-3’	5’-CCCTACACTGGGATGCAGTT-3’
Ucp1	5’-AAACAGAAGGATTGCCGAAA-3’	5’-TGCATTCTGACCTTCACGAC-3’
Cidea	5’-TGCTCTTCTGTATCGCCCAGT-3’	5’-GCCGTGTTAAGGAATCTGCTG-3’
Cox7a	5’-CAGCGTCATGGTCAGTCTGT-3’	5’-AGAAAACCGTGTGGCAGAGA-3’
Cox8b	5’-GAACCATGAAGCCAACGACT-3’	5’-GCGAAGTTCACAGTGGTTCC-3’
Fgfr2	5’-CACTCGGGGATAAATAGCTCCAAT-3’	5’-GCCAAAGTCTGCTATCTTCATCAC-3’
**PCR primers for cloning**		
Fgf10 3'UTR bs1	5’-TCCTCCCCATGACGATCCAA-3’	5’-ATGACCCAAGTGCTTTCCAGT-3’
Fgf10 3'UTR bs2	5’-TGGACCACCCACAACCAAAA-3’	5’-CAGGGGGAAATGTAGGGTGG-3’
Mutated *Fgf10* 3'UTR bs1	5’-TCAAGTTTGGATGGAAGTTATCACG ATGCGAACAATGTTGTGGTGGGGGC-3’	5’-GCCCCCACCACAACATTGTTCGCAT CGTGATAACTTCCATCCAAACTTGA-3’
Mutated *Fgf10* 3'UTR bs2	5’-CTATGTGTAAACAGTCATCACGATAGT ACTGCGGACATTAAACAGCTTCTAGCA-3’	5’-TGCTAGAAGCTGTTTAATGTCCGCAGT ACTATCGTGATGACTGTTTACACATAG-3’
